# Accreditation in general practice in Denmark: study protocol for a cluster-randomized controlled trial

**DOI:** 10.1186/s13063-017-1818-6

**Published:** 2017-02-13

**Authors:** Merethe K. Andersen, Line B. Pedersen, Volkert Siersma, Flemming Bro, Susanne Reventlow, Jens Søndergaard, Marius Brostrøm Kousgaard, Frans B. Waldorff

**Affiliations:** 1 Research Unit of General Practice, Institute of Public Health, University of Southern Denmark, Odense, Denmark; 2 COHERE, Department of Business and Economics, University of Southern Denmark, Odense, Denmark; 3 The Research Unit for General Practice and Section of General Practice, Department of Public Health, University of Copenhagen, Copenhagen, Denmark; 4 The Research Unit for General Practice and Section of General Practice, Department of Public Health, Aarhus University, Aarhus, Denmark

**Keywords:** Accreditation, General practice, Clinical effects, Cluster-randomized trial

## Abstract

**Background:**

Accreditation is used increasingly in health systems worldwide. However, there is a lack of evidence on the effects of accreditation, particularly in general practice. In 2016 a mandatory accreditation scheme was initiated in Denmark, and during a 3-year period all practices, as default, should undergo accreditation according to the Danish Healthcare Quality Program. The aim of this study is primarily to evaluate the effects of a mandatory accreditation scheme.

**Methods/design:**

The study is conducted as a cluster-randomized controlled trial among 1252 practices (clusters) with 2211 general practitioners in Denmark. Practices allocated to accreditation in 2016 serve as the intervention group, and practices allocated to accreditation in 2018 serve as controls. The selected outcomes should meet the following criteria: (1) a high degree of clinical relevance; (2) the possibility to assess changes due to accreditation; (3) availability of data from registers with no self-reporting data. The primary outcome is the number of prescribed drugs in patients older than 65 years. Secondary outcomes are changes in outcomes related to other perspectives of safe medication, good clinical practice and mortality. All outcomes relate to quality indicators included in the Danish Healthcare Quality Program, which is based on general principles for accreditation.

**Discussion:**

The consequences of accreditation and standard-setting processes are generally under-researched, particularly in general practice. This is the largest study in general practice with a randomized implementation approach to evaluate the clinical effects of a nation-wide mandatory accreditation scheme in general practice.

**Trial registration:**

ClinicalTrials.gov, NCT02762240. Registered on 24 May 2016.

**Electronic supplementary material:**

The online version of this article (doi:10.1186/s13063-017-1818-6) contains supplementary material, which is available to authorized users.

## Background

Accreditation is a procedure in which a recognized external institution evaluates an organization on the basis of a predefined set of quality standards. This usually involves a formal site visit by a team of surveyors [1]. The evaluation results in a decision on the granting of accreditation status. Accreditation has become a widespread tool for quality control and improvement in healthcare, and many resources are spent on developing and implementing accreditation systems across the world [2]. Nevertheless, solid evidence for the effects of accreditation is generally lacking, and this is particularly true for general practice [3, 4]. Only two effect studies on accreditation met the quality standards for inclusion in the first Cochrane review [5] on the subject, and none of these studies were about accreditation in general practice. Further, a review on the status of accreditation in primary care concluded that there is a dearth of research on the nature and uptake of accreditation in this sector along with how accreditation affects outcomes of care, and whether it is an effective method to improve quality, perceptions of care, healthcare utilization and costs [3]. In addition to a call for evidence of the clinical effects of accreditation, healthcare professionals have expressed concerns about the bureaucracy and the extra registration work imposed by accreditation.

In 2014 it was decided to implement a mandatory accreditation scheme in Danish general practice in the period 2016–2018. This protocol paper describes the design of a cluster-randomized evaluation of this accreditation scheme by means of the Consolidated Standards of Reporting Trials (CONSORT) extension and the Standard Protocol Items: Recommendations for Interventional Trials (SPIRIT) statement for cluster-randomized trials (Additional files 1 and 2).

## Methods/design

### Participants and study design

The accreditation scheme in Denmark is rolled out as a cluster-randomized controlled trial (CRCT). All 1922 general practices are eligible for the study and are allocated to undergo accreditation in 2016, 2017 and 2018 respectively. Practices allocated to accreditation in 2016 (accreditation2016) and representing 604 clusters are selected as the intervention group, while practices allocated to accreditation in 2018 (accreditation2018) represent 648 clusters and serve as the control group. Practices are assigned an accreditation date 1 year in advance, and in order to avoid contamination from the intervention group, practices allocated to accreditation in 2017 (accreditation2017), representing 664 practices, will be excluded from analysis.

This design, in addition to the comprehensive Danish National Health registers, offers a unique opportunity to conduct research on the effects of accreditation [6–8]. Moreover, the design makes possible a comparison of practices that have already accomplished accreditation with practices waiting to be accredited [9]. Additionally, the national Civil Registration System (CRS) facilitates the collection of relevant data independent of the involved practices [10].

The Danish healthcare system is tax-financed, and most general practitioners (GPs) and hospital services are free of charge. General practice is characterized by five key components. (1) There is a list system associating citizens with a GP. GPs can close for uptake when they have 1600 persons on the list but are allowed to enroll up to 2550 persons. (2) The GP acts as gatekeeper and first-line provider in the sense that a referral from a GP is required for most office-based specialists and always for inpatient and outpatient hospital treatment. (3) An after-hours system is staffed by GPs on a rotation basis. (4) There is a mixed capitation and fee-for-service system. (5) GPs are self-employed, working on contract for the public funder based on a national agreement that details not only services and reimbursement but also opening hours and required postgraduate education [11]. Danish general practice constitutes 1922 practices comprising a total of 3329 GPs.

The accreditation program is developed and managed by the Danish Institute for Quality and Accreditation in Healthcare (IKAS). IKAS offers a range of accreditation programs, tailored for private hospitals, community pharmacies, community healthcare, general practice and specialist physicians practicing outside of a hospital setting.

One year in advance, practices are notified about their accreditation date by letter from IKAS. It is expected that practices will begin working with the standards from that date onwards.

### Randomization

IKAS decided that surveys in all five regions of Denmark should be evenly distributed for the 3 years during which the accreditation process takes place. Moreover, it was decided by IKAS that the accreditation should be conducted by means of municipalities (each Danish region is divided into several smaller municipalities) and that the order of municipalities should be completely random.

**Fig. 1:**
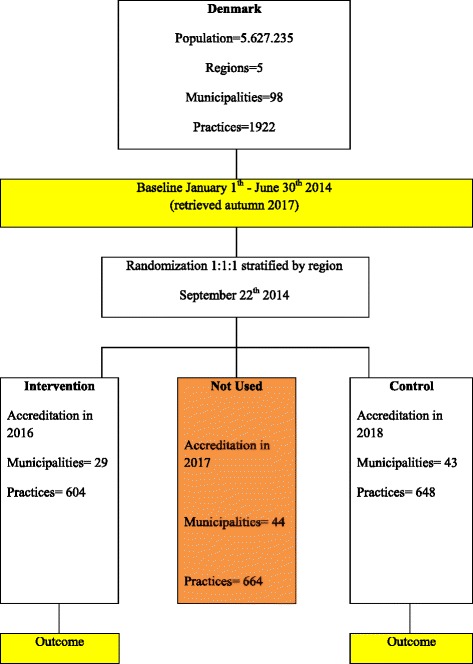
Trial flow for accreditation in general practice (AKIAP)

Time for accreditation of the practices in the 98 municipalities in Denmark was determined in a randomized lots drawing conducted on 22 September 2014 by IKAS, and provided the basis for a prospective study. Two impartial persons from IKAS conducted the drawing. Within each of the five Danish regions, one-third of the practices within the municipalities that were drawn first were allocated to accreditation in 2016, the next third to 2017 and the last third to 2018. If large municipalities were drawn and this resulted in an excess of practices greater than one-third of the practices in a region, the practices were split over 2 allocation years based on the first letter in the street name in ascending order (Fig. 1).

This randomization allows for comparisons between early accredited practices (2016, *n* = 604 practices) and late accredited practices (2018, *n* = 648 practices) in 2017. Every practice is considered a cluster, which means that there are 1252 clusters in the study. The CRCT study is designed to comply with the recommendations of the CONSORT statement: extension to cluster-randomized trials [12] and SPIRIT.

### Intervention

Since 2009 accreditation under the Danish Healthcare Quality Program (DHQP) has been mandatory in the secondary healthcare system in Denmark, and it was decided as part of ’the national strategy for quality development in the healthcare system – common aims and plan of action 2002–2006’. The DHQP has been adjusted to include general practice. The intervention comprises the rollout of a mandatory accreditation scheme, which is defined and carried out by an acknowledged, impartial institution: IKAS.

The DHQP is based on general principles for accreditation. The model contains a set of accreditation standards as well as an accreditation process [13]. The accreditation standards were developed by IKAS in collaboration with representatives from the Organization of General Practitioners in Denmark (PLO), Danish Regions, Danish Patients, and the Danish Association of Practicing Medical Specialists. A preliminary version of the standard set was pilot-tested in 26 practices in 2012 [14]. Subsequently, the standard set was further adjusted, and the Danish Regions and the PLO approved the current version in 2014.

The DHQP for general practice consists of 16 standards with associated indicators within the following areas: (1) quality and patient safety, (2) patient safety critical standards, (3) good patient continuity of care and (4) management and organization [13].

The standards include certain minimal requirements, but are also written to stimulate quality improvement. Not everything in the standards is written to describe and delineate precisely what the client should do. Parts of the standards are intended to stimulate reflection on one’s own practice and thereby inspire improvement activities.

Each standard includes a descriptive part, where the purpose and meaning of the standard is explained in more or less detail, as appropriate. Furthermore, each standard includes a number of indicators that comprise the measurable elements of the standard set. Compliance with the standard set is assessed by rating of the indicators. The surveyor team does this during survey.

A survey implies a visit from a surveyor team consisting of healthcare professionals who have received specific training to handle this task. Some surveyors in the practice sectors are IKAS employees, but all survey teams will include surveyor(s) who are peers, working most of their time in healthcare.

The survey has a dual purpose: to assess compliance with minimal requirements and to identify opportunities for improvement, even when the threshold for obtaining accreditation has been reached. A client needs to comply with the minimal requirements in order to obtain accreditation. But the survey should also give the client feedback on his or her efforts to meet the purpose of the standards, and it should do this in a way that inspires and supports quality improvement work.

Compliance assessment is governed by defined rating principles. The fundamentals are the same for all programs:

- Met: The client in all essentials complies with the requirements in the indicator.
- Largely Met: There are opportunities for improvement, but accreditation can be awarded; no further action is required.
- Partially Met: There are opportunities for improvement, and action is required, unless accreditation is awarded with comments or, in case of critical non-compliances, is denied.
- Not Met: There is no evidence for compliance or only plans to achieve compliance [13].

The standards are accompanied by rating principles, describing how compliance with the indicators is assessed and rated [13].

### Objectives

**Fig. 2:**
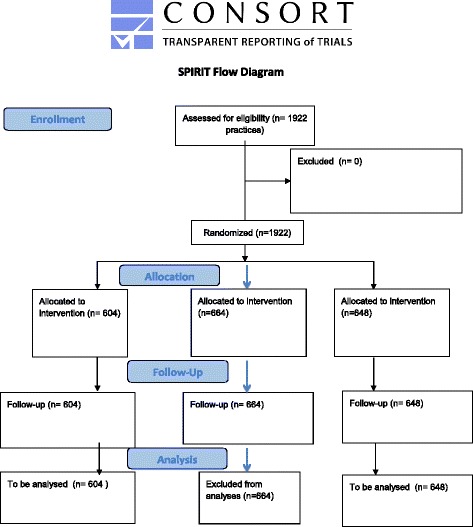
SPIRIT flow diagram for the AKIAP project

The aim of this study is to evaluate the effects of accreditation in general practice with regard to certain clinical outcomes (Fig. 2).

### Clinical outcomes

The selected outcomes should meet the following criteria: (1) a high degree of clinical relevance, (2) the possibility to assess changes due to accreditation, (3) the availability of data independent of self-reporting from practices. Table 1 lists GPs and practice characteristics. The selected outcomes, their origin from the DHQP and the used data collection sources are listed in Table 2.

**Table 1:** GP and practice characteristics distributed on randomization groups

	Accreditation in 2016	Accreditation in 2018	Total	*p* value χ^2^ test
GPs	1106	1105	2211	
Age				
Mean	53.44	52.91		*t* test: 0.64
Gender				0.32
Male	572	548	1120	
	(52%)	(50%)	(51%)	
Female	534	557	1091	
	(48%)	(50%)	(49%)	
Practice type				0.08
Single-handed	314	351	665	
	(28%)	(32%)	(30%)	
Partnership	792	754	1546	
	(72%)	(68%)	(70%)	
Region				0.71
Capital	316	330	646	
	(28%)	(30%)	(29%)	
Central Denmark	272	269	541	
	(25%)	(24%)	(25%)	
North Denmark	113	94	207	
	(10%)	(9%)	(9%)	
Zealand	155	157	312	
	(14%)	(14%)	(14%)	
Southern Denmark	250	255	505	
	(23%)	(23%)	(23%)

**Table 2:** Description of outcomes according to DHQP standards and data sources

Primary outcome	DHQP standard ’aim’	Rationale	Data source
Changes in number of prescribed drugs in patients older than 65 years in period	2.2. Prescriptions and prescription renewal: *“Physicians in the practice have knowledge about the basic list for rational pharmacotherapy. The practice conducts, according to medical judgments, annual controls in patients with chronic disease, comprising assessment of prescriptions”.*	Inappropriate use of many concurrent drugs, especially in older people, imposes a substantial burden of adverse drug events, ill health, disability, hospitalization, and even death. National guidelines for general practice recommend critical reviews of the elderly patients’ medication, and secure medication in elderly patients relates to all four areas in DHQP, but most of all it relates to the patient security critical standards	Medication Database (MD)
Secondary outcomes			
Polypharmacy in aged patients >65 years Polypharmacy Yes/No	2.2. Prescriptions and prescription renewal: *“Physicians in the practice have knowledge about the basic list for rational pharmacotherapy. The practice conducts, according to medical judgements, annual controls in patients with chronic disease, comprising assessment of prescriptions”.*	National guidelines for general practice recommend critical reviews of the elderly patients’ medication (18)(18)(18)(15)(19)(18), and secure medication in elderly patients relates to all four areas in DHQP, but most of all it relates to the patient security critical standards. This variable is the dichotomized primary outcome defining polypharmacy as more than 5 concurrent prescribed medications.	Medication Database (MD)
Patients >65 years taking NSAIDs without a concurrent prescription for proton pump inhibitor (PPI) Daily drug dose (DDD) of NSAID without PPI in period	2.2. Prescriptions and prescription renewal: *“Physicians in the practice have knowledge about the basic list for rational pharmacotherapy. The practice conducts, according to medical judgements, annual controls in patients with chronic disease, comprising assessment of prescriptions”.*	Non-steroidal anti-inflammatory drugs (NSAIDs) can cause serious gastrointestinal complications, and it is estimated that more than 100 persons die from NSAID-induced gastrointestinal bleeding and perforation in Denmark annually. The risk is higher in the elderly. PPIs reduce the prevalence of bleeding gastric ulcers and reduce the risk of dyspepsia and uncomplicated gastric ulcers in NSAID treatment. Therefore, PPI treatment is recommended in combination with NSAIDs where these are requisite in patients older than 65 years of age.	Medication Database (MD)
Sleeping medication Indicator: reduction in DDD sleeping medication/1000 patients DDD sleeping medicine in period	2.2. Prescriptions and prescription renewal: *“Physicians in the practice have knowledge about the basic list for rational pharmacotherapy. The practice conducts, according to medical judgements, annual controls in patients with chronic disease, comprising assessment of prescriptions”.*	National guidelines from the National Board of Health recommend reduction in the prescription of sleeping medication.	Medication Database (MD)
Preventive home visits Indicator: Changes in percentage of patients >75 years who have had a preventive home visit Preventive home visit Yes/No	1.2. Use of good clinical practice – vulnerable patient groups: *“Patients are diagnosed, treated and provided support for self care and they are controlled, referred and rehabilitated in accordance with good clinical practice”*	The care of fragile elderly has a high priority and is addressed in this standard. Moreover, a specific fee for conducting preventive home visits to fragile elderly is part of the general practice remuneration system.	The Danish National Health Services Register
Annual controls for chronic disease Indicator: changes in number of conducted annual controls for chronic disease/1000 patients Number of annual controls in period	1.2. Use of good clinical practice – chronic disease: *“Patients are diagnosed, treated and provided support for self care and they are controlled, referred and rehabilitated in accordance with good clinical practice”*	Danish GPs coordinate most of chronic disease management and conduct annual controls. Remuneration for annual control is possible, only once a year, for certain chronic diseases: diabetes, psychiatric disease, cardiovascular disease (CVD), osteoporosis, chronic obstructive pulmonary disease (COPD), musculoskeletal disease, dementia and cancer.	The Danish National Health Services Register
Spirometry in COPD/asthma Indicator: changes in number of conducted spirometry/1000 patients Spirometry Yes/No in period	1.2. Use of good clinical practice – chronic disease: *“Patients are diagnosed, treated and provided support for self-care and they are controlled, referred and rehabilitated in accordance with good clinical practice”*	COPD and asthma patients should be monitored on an annual base with spirometry. GPs are remunerated with a certain fee for conducting spirometry.	The Danish National Health Services Register
Number of reported adverse events (RAEs) Indicator: changes in number of RAEs RAE Yes/No in period per practice	1.3. Reported adverse events – the aim of the standard is to: “*Reduce the risk of patient injuries following adverse events and to create learning and improvement on the background of reported adverse events”.*	Reporting of accidental events is addressed in standard 1.3. It is not only the aim to reduce the *incidence* of accidental events, but also to improve the management and learning potential of accidental events. Consequently, a simple count of RAEs may be equivocal. In order to avoid bias, we only analyse if at least one RAE has been reported from a practice in period.	Danish Patient Safety Database (DPSD)
Patient satisfaction survey Changes in proportion of practices with a patient satisfaction survey in period	1.4. Patient evaluations – the aim of the standard is to: *“Generate learning and improve the clinic’s services on the basis of patient feedback”.*	Patient-experienced quality will be surveyed using data from DANPEP (Danish Patients Evaluate Practice) (23, 24). DANPEP is a nationwide, continuous assessment of patient contentment in general practice. We only evaluate if a patient satisfaction survey is conducted in period.	DANPEP database (Danish Patients Evaluate Practice).
Mortality Indicator: changes in mortality rates (deaths/1000 inhabitants) Number of deaths in 2-month period after index date		Mortality is the ultimate measure for effects of all applied interventions in health sciences. Any changes in mortality rates in relation to accreditation are of interest and should be a focus for analysis. Data on mortality rates can be obtained from (SD).	Danish Register of Causes of Death

*SD* Statistics Denmark (StatDen)

#### Primary outcome

The primary outcome is the changes in number of prescribed drugs in patients older than 65 years between the baseline and follow-up period.

This outcome refers to standard 2.2 in the DHQP: physicians in the practice have knowledge about the basic list for rational pharmacotherapy. The practice conducts, according to medical judgments, annual controls in patients with chronic disease, comprising assessment of prescriptions.

The primary outcome is selected because inappropriate use of many concurrent drugs, especially in older people, imposes a substantial burden of adverse drug events, ill health, disability, hospitalization and even death. The single most important predictor of inappropriate prescribing adverse drug events (ADEs) in older patients is the number of prescribed drugs [15]. One report estimated the risk of ADE as 13% for two drugs, 38% for four drugs and 82% for seven drugs or more [15]. A Danish study from 2000 showed that among 75-year-old persons living at home, 60% used three or more prescribed drugs and 34% used five or more [16]. A more recent study performed by the pharmacist foundation found that almost 40% of the elderly older than 65 years use six or more drugs, while this is the case for more than 50% of the elderly older than 80 years [17]. National guidelines for general practice recommend critical reviews of the elderly patients’ medication [18], and secure medication in elderly patients relates to all four areas in DHQP.

It is not possible to assess whether the medication given to the individual patient is reasonable, as we have no access to the patients’ diagnosis or the indication. Therefore, we will analyse any changes in number of drugs in elderly persons (older than 65) for accredited practices and non-accredited practices.

Our main hypothesis is that implementing DHQP will decrease the number of prescribed drugs during the accreditation scheme.

#### Secondary outcomes

The secondary outcomes focus on safe medication practices, good clinical practices and quality and patient safety according to the DHQP. These outcomes relate directly to one of the standards concerning critical patient safety issues (standard 2.2), and they are expressed as changes in amounts or prevalence between the observation period and the baseline period.

The rationale for each of the secondary outcomes is presented in Table 2.

##### Safe medication

Safe medication use includes:

- Changes in the proportion of polypharmacy (>5 prescribed drugs) patients older than 65 years
- Changes in daily drug dose (DDD) of non-steroidal anti-inflammatory drugs (NSAIDs) without proton pump inhibitor (PPI)
- Changes in DDD of sleeping medicine.

##### Good clinical practice

Good clinical practice includes:

- Changes in the proportion of elderly persons older than 75 receiving a preventive home visit (PHV)
- Changes in the number of annual controls for chronic disease (ACCD) between periods
- Changes in the number of spirometries performed
- Quality and patient safety:
- Changes in proportion of practices with a reported adverse event (RAE)
- Changes in proportion of practices with a patient satisfaction survey between periods.

##### Mortality

Mortality is evaluated as changes in mortality rates between periods.

### Data collecting periods

All outcomes except mortality are surveyed in the following two periods:

1. Baseline data are collected from 1 January 2014 to 30 June 2014.
2. Follow-up data are collected from 1 January 2017 to 30 June 2017.

For mortality the periods are:

1. Baseline data are collected from 30 June2014 to 31 August 2014.
2. Follow-up data are collected from 30 June 2017 to 31 August 2017.

### Data sources

Data on clinical effects measures are prospectively collected and stored within various national registers. However, the project group does not have permission to retrieve data until autumn 2017. Prescription data from 2015 formed the basis for the power analysis. Because of the CRS, these data can be linked to the patients’ civil registration number and analysed within a common research database hosted by Statistics Denmark (StatDen) and Statens Serum Institut (SSI). A named responsible statistician will obtain permission to work with data within the frames of StatDen.

We will use the National Prescription Register (NPR), which is a database that includes records of all prescriptions dispensed at all Danish community pharmacies since 1995 (information on drugs administered to hospital inpatients is not registered) to calculate the number of drugs per patients based on the date a prescription is redeemed, the pharmaceutical group (ATC code 4) and the DDD in the redeemed prescription. Based on this information, we will calculate the number of pharmaceutical groups through which an individual redeemed prescriptions on 30 June in the years 2014 and 2017.

The Danish Register of Causes of Death (DRCD), which includes information on specific causes of death based on death certificates, will be used to define all-cause mortality.

GP and practice characteristics and services provided (e.g. spirometry and annual check-ups for chronic conditions) will be retrieved from the Danish National Health Services Register (DNHSR).

All baseline data are available in the mentioned registers/databases and will be retrieved starting from autumn 2017.

### Statistics

Differences in baseline characteristics are reported as numbers (percentages) and tested using chi-squared tests.

Linear regression will be used for continuously valued outcomes (e.g. number of different drugs, safe medication, annual controls, etc.) and logistic regression for binary outcomes (polypharmacy, preventive home visits, changes in proportion of practices with reported adverse events and changes in proportion of practices with a patient satisfaction survey). Analyses will account for clustering of patients in practices by generalized estimating equations (GEE). A level of 5% will be taken as statistically significant.

Analyses will be performed in SAS version 9.4 (SAS Institute, Cary, NC, USA).

### Power calculation

The mean number of Danes older than 65 years allocated to an average practice is 584 (standard deviation (SD) 378; registry data at the start of 2015). The mean number of redeemed prescriptions per patient among Danes older than 65 years is 6.59 (SD 5.04; registry data over the full year 2014). For the power calculation, the intra-class correlation (ICC) is set relatively high at 0.2.

Using the formulae in Hemming et al. [19], we are able with our data to detect a difference from a mean number of prescriptions of 6.59 in the control group to 6.13 in the accreditation group using a *t* test with a 5% level of significance accounting for clustering within practices with a power of 80%.

### Trial status

Randomization has been conducted. Power calculations are based on prescription data from 2015. All practices and GPs have been recruited. Data on clinical effects measures are prospectively collected and stored within various national registers. However, the project group does not have permission to retrieve data until autumn 2017.

## Discussion

The consequences of accreditation and standard-setting processes are generally under-researched; this is particularly true in general practice. This paper describes the rationale and design of the first cluster-randomized controlled trial to evaluate the clinical effects of a nation-wide accreditation scheme in general practice, focusing on the effects of accreditation on changes in the number of prescribed drugs in patients older than 65 years. Our study is in accordance with several recent calls for more research into the clinical effects of accreditation in healthcare [3, 5, 9, 20, 21]. This is the first study with a CRCT approach to measure clinical effects of accreditation in general practice. Thus, the knowledge emanating from this study will be unique, and the study may set a benchmark for more research.

The study is an effectiveness study based on register data, and thus is not dependent on inputs from participants or their willingness to participate; hence, the occurrence of information and selection bias is reduced. Moreover, the randomization seems to be successful, as GPs and their practices do not differ between accreditation years.

Randomization group sizes were dependent on allocation and determined by IKAS; thus, this decision was out of the hands of the project group. Allocation was conducted by draw, and the persons who drew lots were impartial. The decision on excluding practices allocated to accreditation in 2017 from the analysis was logical and pragmatic, as any spillover effect from the intervention group accreditation year 2016 is suggested to be largest for accreditation year 2017 and least for accreditation year 2018.

Experiences from the French healthcare system suggest that accreditation risks being reduced to a matter of standardizing practices rather than a matter of improving quality [22]. Therefore, our study primarily focuses on clinically relevant quality indicators relating to the DHQP. The research group scrutinized the standards in the accreditation scheme, and the final list of endpoints was established according to relevant outcomes from a clinical perspective. The direction of changes for some of the chosen outcomes is ambiguous; e.g. polypharmacy can be appropriate in patients with multi-morbidity, but it may also be a result of a lack of treatment plans and medicine reviews. However, according to several national guidelines and systematic reviews, polypharmacy should be reduced, especially among the elderly.

Because the accreditation scheme is mandatory for all Danish GPs, the results are expected to be representative for the effects of accreditation in Danish general practice. Extrapolation to other countries requires further consideration due to differences in the organization of healthcare systems and cultural factors.

The Danish GPs have been informed about the forthcoming accreditation at least 12 months ahead. It may be assumed that some practices will prepare their accreditation in good time as part of their practice development work. Further, some spillover effects must be expected from practices undergoing accreditation to practices awaiting accreditation. Therefore, we collect baseline data from the first half of 2014, and leave out the 2017 population in our analysis.

The GPs allocated to the three clusters did not differ statistically significantly in gender, practice type or region. Therefore, we assume that eventual changes in performance cannot be ascribed to selection.

## Additional files

Additional file 1: CONSORT extension for cluster trials checklist. (DOCX 36 kb)
Additional file 2: SPIRIT checklist. (DOC 121 kb)

## Abbreviations

ACCD: Annual controls for chronic disease
ADE: Adverse drug event
AKIAP: Accreditation in general practice
COPD: Chronic obstructive pulmonary disease
CRS: Civil Registration System
DANPEP: Danish Patients Evaluate Practice
DDD: Daily drug dose
DHQP: Danish Healthcare Quality Programme
DNHSR: Danish National Health Services Register
DPSD: Danish Patient Safety Database
DRCD: Danish Register of Causes of Death
GP: General practitioner
IKAS: Danish Institute for Quality and Accreditation in Healthcare
MD: Medication Database
NPR: National Prescription Register
NSAID: Non-steroidal anti-inflammatory drug
PHV: Preventive home visit
PLO: Organization of General Practitioners in Denmark
PPI: Proton pump inhibitor
RAE: Reported adverse event
SD: Standard deviation
SSI: Statens Serum Institut
StatDen: Statistics Denmark
